# Myopia induces meridional growth asymmetry of the retina: a pilot study using wide-field swept-source OCT

**DOI:** 10.1038/s41598-020-67940-4

**Published:** 2020-07-02

**Authors:** Katharina Breher, Arne Ohlendorf, Siegfried Wahl

**Affiliations:** 1 Institute for Ophthalmic Research, Eberhard Karls University Tuebingen, 72076 Tübingen, Germany; 2 Carl Zeiss Vision International GmbH, 73430 Aalen, Germany

**Keywords:** Imaging and sensing, Computed tomography, Predictive markers

## Abstract

Myopic axial eye growth has mechanical implications on ocular structures, such as the retinal and foveal shape integrity or choroidal thickness. The current study investigated myopia-related changes of retinal radius of curvature, foveal width, depth, slope and choroidal thickness. Wide-field swept-source OCT line and volume scans were performed on 40 young adult participants in horizontal and vertical directions. OCT scans were corrected for their scan display distortions before automated extraction of retinal and foveal shape parameters. All findings were correlated to refractive error and axial length. The horizontal retinal radius of curvature and the directional ratio between horizontal and vertical retinal shape correlated significantly with axial length ($\rho =+0.53, p<0.001$ and $\rho =+0.35, p<0.05$). Vertical retinal shape and foveal pit parameters neither showed any significant correlations with axial length nor refractive error (all $p>$ 0.05). Choroidal thickness correlated significantly with refractive error in all analyzed regions ($\rho +0.39\,\mathrm{to}\,+0.52$), but less with axial length ($\rho -0.18$ to − 0.37). Horizontal retinal shape and choroidal thickness, but not foveal pit morphology, were altered by myopic eye growth. Asymmetries in horizontal versus vertical retinal shape with increasing myopia were detected. These parameters could act as promising biomarkers for myopia and its associated complications.

## Introduction

Myopia is a worldwide problem with rising prevalence, affecting half of the population in 2050^1^. Myopia is caused by a mismatch between the ocular focal length and axial length due to excessive eye growth^2^. An increased myopic refractive error thus reflects a longer axial length, which leads to growth-induced retinal changes due to mechanical stretching of the tissue. Therefore, it appears logical that myopia also increases the risk especially for retinal pathologies, such as myopic maculopathy and retinal detachment^3,4^. There is a dose–response relationship, which indicates a proportional relationship between amplitude of the refractive error and the risk to be affect by a particular complication. Myopes with a refractive error of − 3D, − 6D and − 9D have a 3-fold, 9-fold and 22-fold higher odds ratio to suffer from retinal detachment, respectively^4,5^. Furthermore, macular degeneration occurs up to 350 times more often in myopes^4,6^. As these diseases commonly lead to a potentially irreversible loss of sight, but also will affect an increasing part of the worldwide population, one aim in myopia research is to identify biomarkers for myopia. Ideally, these biomarkers should describe myopic eye growth and associated risks, while being obtained by clinically feasible measurement procedures. Since the retina is one of the most affected tissues during eye growth, the investigation of retinal shape and layer thickness has become a subject of interest in myopia research.

Retinal shape can be measured either directly using magnetic resonance imaging (MRI)^7^, but also indirectly via peripheral biometry^8–11^ or peripheral refraction^12–14^. The retinal shape can then be inferred from the differences between eccentric and central refraction or axial length. However, indirect conclusions from eccentric eye length or peripheral refraction should only be made cautiously. Optical aberrations occur in off-axis measurement angles, which are caused by eye, instrument or head rotations, and which cannot be predicted and thus considered in the individual subjects^15–17^. Therefore, indirect measurement techniques suffer from a certain inaccuracy, while recent direct procedures, such as MRI, are of reduced clinical feasibility and too cost-intensive. Due to these limitations, optical coherence tomography (OCT) as established tool for in-vivo retinal imaging was taken into account for the analysis of retinal shape. However, original OCT scan images appear flattened due to displaying reasons. These displaying distortions can be corrected through optical re-modelling^18–21^. Consequently, the retinal shape can be extracted from these distortion-corrected OCT scan images.

Additionally, foveal pit morphology—such as width, depth and slope—can be analyzed via OCT imaging either with original^22–28^ or corrected scans^29^.

Choroidal thickness presents another potentially important biomarker for myopia. Multiple studies investigated the choroid in relation to refractive error and axial length. They used a broad variety of methodological tools regarding OCT technology (spectral-domain vs. swept-source OCT), segmentation method (manual vs. automated) and evaluated locations (single point locations vs. broader areas). The majority finds decreasing choroidal thickness with increasing myopia^30–35^.

In summary, it is of high interest to determine if and which ocular parameters—additionally to axial length and refractive error—could be used to describe and evaluate myopic eye growth. So far, retinal shape has been measured mostly via eccentric biometry, refraction or MRI with the known limitations. Additionally, retinal shape was investigated using corrected spectral-domain OCT scans but with a relatively small scan field. The relation of foveal pit shape to refractive error has not been investigated yet with distortion-corrected scans. In contrast, the purpose of previous studies was choroidal thickness and correlations to axial length or refractive error. However, these groups used spectral-domain OCT with associated reduced visibility of the choroid, manual and potentially biased segmentation methods or smaller scan fields.

The current study was thus focused to identify whether distortion-corrected retinal shape, foveal pit morphology, as well as choroidal thickness exhibit correlations with axial length or refractive error. If this is the case, these parameters could figure as appropriate biomarkers for myopia, measurable in a clinically feasible setting using OCT and biometry.

## Results

### Normative values

Table 1 shows an overview about the averages and distribution of the analyzed parameters in all scan directions (retinal and foveal morphology) and sectors (choroidal thickness). Data are presented as median and interquartile range (IQR). In total, the horizontal retinal radius of curvature is smaller than the vertical, leading to a horizontal-to-vertical ratio of < 1.00 between both meridians. The same proportion can be found for foveal depth and slope, where the vertical values exceed the horizontal values and a subsequent ratio of < 1.00. Foveal width presents an exception, as the horizontal width is larger than the vertical width, resulting in an average directional relation of > 1.00. Choroidal thickness is generally thicker in the central than peripheral retina. Moreover, regional differences can be detected, with thicker choroids in the superior and temporal areas compared to inferior and nasal areas.

**Table 1:** Median and IQR values for the investigated paramters.

	Median ± IQR (mm/$^\circ$)		Median ± IQR ($\upmu$m)
RRC H	13.20 ± 1.80	ChT #1	379 ± 119
RRC V	13.32 ± 2.63	ChT #2	381 ± 144
RRC H/V	0.98 ± 0.11	ChT #3	367 ± 127
Foveal width H	1.21 ± 0.25	ChT #4	382 ± 122
Foveal width V	1.16 ± 0.25	ChT #5	349 ± 143
Foveal width H/V	1.08 ± 0.10	ChT #6	363 ± 131
Foveal depth H	0.11 ± 0.03	ChT #7	351 ± 128
Foveal depth V	0.12 ± 0.03	ChT #8	357 ± 104
Foveal depth H/V	0.94 ± 0.11	ChT #9	281 ± 126
Foveal slope H	14.00 ± 3.97	ChT #10	340 ± 98
Foveal slope V	16.54 ± 3.51	ChT #11	290 ± 88
Foveal slope H/V	0.89 ± 0.11	ChT #12	313 ± 66
		ChT #13	213 ± 82

RRC, retinal radius of curvature; H, horizontal scan meridian; V, vertical scan meridian; ChT #n, choroidal thickness in specific ETDRS area

### Correlations to axial length and refractive error

**Figure 1:**
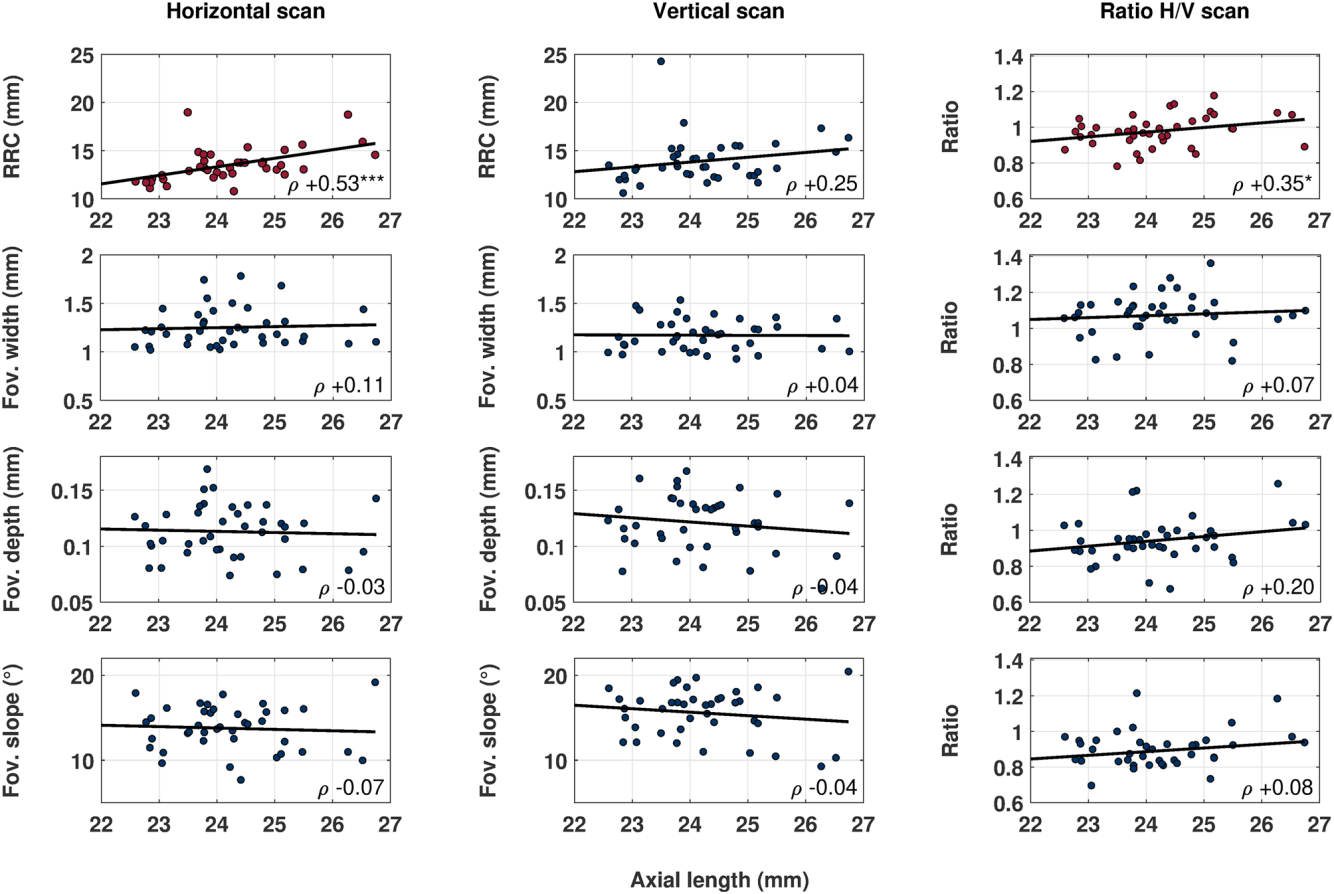
Correlations of axial length with retinal and foveal shape parameters in horizontal and vertical scan meridians, as well as their H/V ratio. For a fast overview, significant correlations with axial length—as in the case with horizontal retinal radius of curvature (RRC), as well as horizontal-to-vertical ratio—are plotted with red markers Asterisks behind the Spearman $\rho$ value denote significant correlations of the retinal parameters with axial length, with *p < 0.05, **p < 0.01, ***p < 0.001.

However, the IQR indicates high intersubject variability in all investigated parameters, which could be based on a dependency of the retinal parameters on axial length or refractive error. Therefore, the retinal and foveal morphology, and choroidal thickness were correlated to axial length and refractive error as a next step, as seen in Table 2 and Fig. 1. Choroidal thickness correlated more often and more significantly with refractive error than axial length. Retinal shape showed significant positive correlations in the horizontal direction ($\rho =+$0.53, *p* < 0.001) and in the directional ratio ($\rho =+$0.35, *p* < 0.05) with axial length, but not in the vertical scan meridian. Foveal width, depth and slope could be associated with neither axial length nor refractive error in any scan direction. Moreover, axial length and refractive error were correlated negatively with each other ($\rho =-$0.71, p < 0.001).

**Table 2:** Spearman correlation coefficient $\rho$ of horizontal and vertical retinal radius of curvature, foveal pit morphology, choroidal thickness and the ratios of horizontal and vertical parameters, with axial length and refractive error, respectively.

	Axial length	Refractive error		Axial length	Refractive error
RRC H	+ 0.53***	$-$ 0.16	ChT #1	$-$ 0.27	+ 0.44**
RRC V	+ 0.25	$-$ 0.01	ChT #2	$-$ 0.35*	+ 0.51***
RRC H/V	+ 0.35*	$-$ 0.27	ChT #3	$-$ 0.29	+ 0.41**
Fov. width H	+ 0.11	$-$ 0.09	ChT #4	$-$ 0.29	+ 0.49**
Fov. width V	+ 0.04	+ 0.13	ChT #5	$-$ 0.32*	+ 0.45**
Fov. width H/V	+ 0.07	$-$ 0.14	ChT #6	$-$ 0.27	+ 0.46**
Fov. depth H	$-$ 0.03	+ 0.10	ChT #7	$-$ 0.28	+ 0.43**
Fov. depth V	$-$ 0.04	+ 0.15	ChT #8	$-$ 0.37*	+ 0.52***
Fov. depth H/V	+ 0.20	$-$ 0.19	ChT #9	$-$ 0.36*	+ 0.46**
Fov. slope H	$-$ 0.07	+ 0.15	ChT #10	$-$ 0.18	+ 0.46**
Fov. slope V	$-$ 0.04	+ 0.07	ChT #11	$-$ 0.20	+ 0.42**
Fov. slope H/V	+ 0.08	+ 0.05	ChT #12	$-$ 0.19	+ 0.46**
			ChT #13	$-$ 0.28	+ 0.39*

RRC, retinal radius of curvature; H, horizontal scan meridian; V, vertical scan meridian; ChT #n, choroidal thickness in specific ETDRS area

Asterisks behind the Spearman $\rho$ value denote significant correlations of the retinal parameters with axial length or refractive error, with *p < 0.05, **p < 0.01, ***p < 0.001.

## Discussion

The current study investigated the influence of myopia—characterized by axial length and spherical refractive error—on retinal shape, foveal pit morphology and choroidal thickness in young adults. In-vivo retinal imaging was performed using wide-field swept-source OCT with distortion-correction of the arrangement of the OCT scan^18^. The findings can be summarized and interpreted as follows:

All retinal radii fall within commonly reported previous results of 8–21 mm^19,20^ and averages between 11 mm and 14 mm^36^. The horizontal retinal radius, as well as the horizontal-to-vertical ratio of radii, revealed significant positive correlations with axial length. Myopes tend to have slightly larger radii and directional ratios than emmetropes. These findings go along with a recent OCT study with a similar methodology^21^. This means, that the common relationship of larger vertical than horizontal radii is not maintained after myopic eye growth. The same directional disparity was also reported for ocular shape in MRI scans^15,37^. Therefore, ocular and retinal shape both change differently in the horizontal and vertical direction. This asymmetrical and changed growth pattern could be also related to a more irregular retinal pigment epithelium (RPE) surface in myopes^38^ and an altered fundus curvature with myopic complications, such as myopic choroidal neovascularization, chorio-retinal atrophy and staphyloma^39^.

Regarding foveal pit morphology, myopes tend to have smaller foveal widths, depths and shallower slopes. However, no statistically significant differences and correlations were shown. This finding is in concordance with an earlier study that indirectly concluded foveal pit shape from uncorrected retinal thickness maps^23^.

Choroidal thickness is generally known to thin with increasing retinal eccentricity, axial length and myopic refractive error, which could be replicated in the current study. Spearman analysis revealed correlation coefficients between $+$ 0.39 and $+$ 0.52 for refractive error, and − 0.18 and − 0.37 for axial length, which is generally in accordance with past studies^30–35^. However, it seems noteworthy that choroidal thickness correlated more with refractive error than axial length, which was an unexpected finding, as it is commonly described vice versa^30,32,34,35^. This difference might be caused by the choice of using the spherical refractive error instead of the spherical equivalent refractive error for the correlations. This way of analysis rules out the influence of astigmatism on correlation results, as astigmatism otherwise would artificially increase the myopic refractive error. Moreover, the choroidal thinning seems to be more attenuated in the central than in the peripheral retina, as well as superiorly more than nasally, as already described earlier^40,41^. Possible relations of these spatial characteristics with different regional choroidal thickness changes in response to defocus^42–44^ need further investigation.

The current study also faces some potential limitations. Firstly, the amount of participants was relatively small, as it was carried out as a pilot-study. Secondly, the majority of the dataset consisted of emmetropic and low to moderate myopic participants, lacking high myopes ($<-$ 6D). In high myopia, more obvious myopia-related changes might have been detected. This could have also been the case if bigger scan angles were used. Although the scan field can be enlarged via add-on lenses or image stitching, this measurement procedure cannot be translated into a feasible clinical setting with patients so far. Moreover, the optical correction model cannot (yet) account for the unpredictable high optical off-axis measurement phenomena, resulting in eventually large inaccuracies in distortion-correction. The used swept-source OCT device provides a maximum of 16 mm line scans and 12 $\times$ 12 mm$^2$, which is approximately double the scan size as in commonly used spectral-domain OCTs. Furthermore, swept-source technology enables a faster scanning procedure with more A-scans and B-scans. The relation of scan time and gain of information becomes of importance when examining patients with reduced fixation ability, due to young age or ocular diseases, as it appears with myopia. In addition, the relatively large scan angle, high resolution and scan depth provide easily visible extra-macular imaging in deep retinal structures. This information is not only needed for the evaluation of retinal shape and choroidal thickness, but also clinical exams for peripheral and choroidal complications in myopia^3,4^.

**Figure 2:**
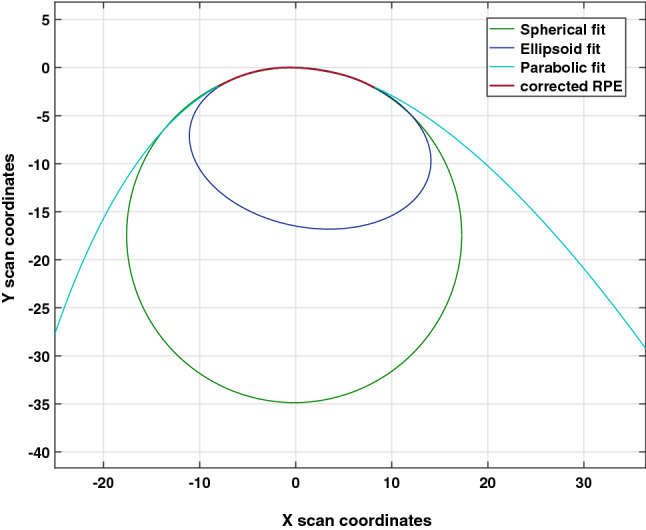
Comparison of different mathematical models to fit the distortion-corrected RPE. Spherical, ellipsoid and parabolic shapes were fitted to the distortion-corrected retina of the 16 mm line scan. Up to the current scan size, none of the models shows a superior fit than the others.

Regarding retinal shape calculations, spherical fits were chosen over ellipsoid fits^7,11,17,20^ or cubic spline functions^21^. This approach was based on two main points: Firstly, a spherical fit does not produce higher fitting errors up to a scan size of 16 mm, as shown with an example OCT scan in Fig. 2. Therefore, it is adequate and accurate compared to more complex models. Secondly, the retinal radius of curvature represents a single and conclusive parameter for the numerical description of retinal shape. Providing short and informative values becomes of high importance when translating and applying research methods and findings into clinical settings.

Furthermore, only line scans were corrected for scan image distortions but not volume scans—from which the choroidal thickness maps were extracted. The distortion-correction of whole cube scans is very time- and memory-extensive with current software and hardware. Also, previous studies showed that absolute errors in uncorrected versus corrected volume scans are in the range of 2.5 $\upmu$m and 6.7 $\upmu$m in the center and in the periphery of the scan field, respectively^19^. Moreover, some peripheral and potentially erroneous choroidal thickness values were not considered in the data analysis, due to the division into Early Treatment of Diabetic Retinopathy Study (ETDRS)^45^ areas. This additional step of distortion-correction was thus skipped, while still maintaining high accuracy.

To conclude, the study provided evidence that changes in choroidal thickness, horizontal retinal radius of curvature and the horizontal-vertical growth ratio exhibit significant correlations to axial length and/or refractive error. Therefore, retinal shape and choroidal thickness, but not foveal pit morphology, are altered by myopia-induced eye growth. Subsequently, these parameters are able to indicate retinal changes caused by eye growth, thus, could figure as promising biomarkers for myopia and associated complications. Moreover, the study provides a fast and feasible measurement procedure and thus an easy translation into clinical practice by using commonly available technology. Longitudinal studies are needed to evaluate the ongoing growth process of retinal, foveal and choroidal parameters in children but also in highly myopic participants with and without myopia-related ocular pathologies.

## Methods

### Study participants

This prospective study was carried out at the Institute for Ophthalmic Research at the University Tuebingen, followed the Declaration of Helsinki and data protection regulations and was approved by the ethics committee of the Faculty of Medicine of the University Tuebingen. Written informed consent was obtained from every study participant prior to the measurements. Participants with ocular pathologies, surgeries, hyperopia or insufficient OCT signal strength < 6 were excluded. In total, 40 young adults (12 males and 28 females) with a mean age of $24.5\pm 3.5$ years were included in the study. Mean refractive error and axial lengths were $-1.33\pm 1.83$D (range $+$ 0.60D to − 5.33D) and $24.22\pm 1.02$ mm (range 22.59–26.74 mm), respectively.

### Instrumentation and measurement procedure

Each participant underwent objective refraction using wavefront aberrometry (ZEISS i.Profiler plus, Carl Zeiss Vision GmbH, Aalen, Germany), ocular biometry (ZEISS IOLMaster 700, Carl Zeiss Meditec AG, Jena, Germany), as well as retinal imaging using swept-source OCT (ZEISS PlexElite 9000, Carl Zeiss Meditec Inc., Dublin, CA, USA). The swept-source OCT system uses a wavelength between 1,040 and 1,060 nm and therefore reaches a scan depth of 3 mm with an axial resolution of 6.3 $\upmu$m in tissue. The OCT scan included different scan patterns: a 12 $\times$ 12 mm$^2$ macular volume scan consisting of 1024 B-scans with 1024 A-scans per B-scan, as well as a horizontal 0$^\circ$ and nearly vertical 80$^\circ$ 16 mm line scan centered on the fovea. The scan meridian of 80$^\circ$ instead of exactly 90$^\circ$ was chosen due to technical limitations in the size of the vertical scan field of the OCT device. The overall OCT field of view is rectangular instead of squared, being larger in the horizontal than vertical dimension. Therefore, a 16 mm line scan would exceed the vertical dimensions of the scan field if oriented 90$^\circ$, in contrast to an 80$^\circ$ directional scan.

### Distortion correction and extraction of retinal morphology parameters

#### OCT distortion correction

**Figure 3:**
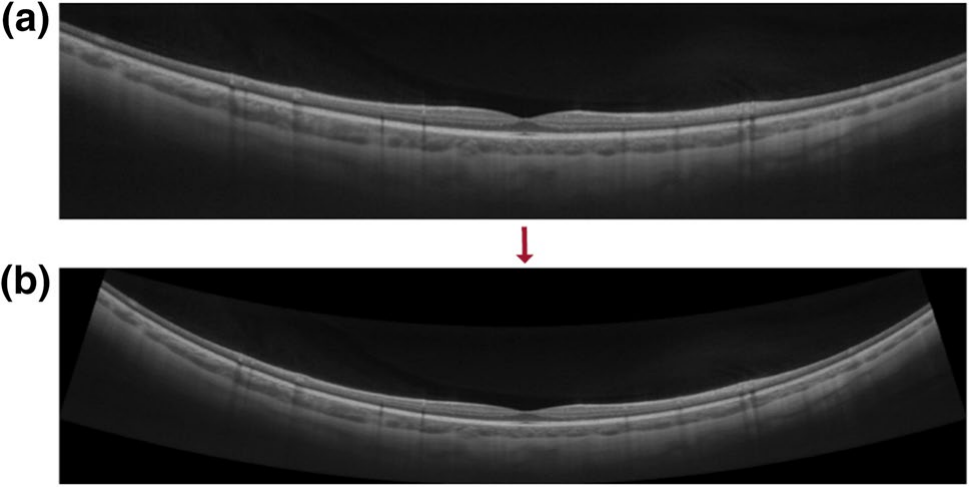
(**a**) Uncorrected, “original” OCT B-Scan of 16 mm length in 80$^\circ$ scan orientation. (**b**) Output B-scan after distortion-correction. Note the increased curvature compared to the artificially flattened, original scan image in (**a**).

Prior to the calculation of the retinal shape and foveal pit morphology, the OCT line scans were corrected for the display distortions as previously validated^18^. Briefly, this approach ray-traces the single A-scan pathways of one B-scan through the optics of the OCT device and ocular surfaces (OpticStudio, Zemax, LLC, Kirkland, WA, USA), resulting in a line of constant group delay for all the A-scans of this particular B-scan. Based on the position and displacement of the line of constant group delay, the scan image is accordingly re-arranged and corrected for the dimensional distortions. Individual eye and scan parameters can be modified in the computer model, e.g. the participants’ individual axial lengths, as done in the current study. An example OCT scan before and after distortion correction is illustrated in Fig. 3.

#### Retinal radius of curvature

After distortion correction, the retinal radius of curvature was determined by fitting a sphere to the distortion-corrected RPE of the B-scans^18^.

#### Foveal pit morphology

Foveal pit parameters were extracted using a previously validated and semi-automated approach^29^. Foveal width, depth and slope were obtained from defined mathematical landmarks of a Sum of Gaussian fit to the foveal surface of the distortion-corrected scan image.

#### Choroidal thickness

**Figure 4:**
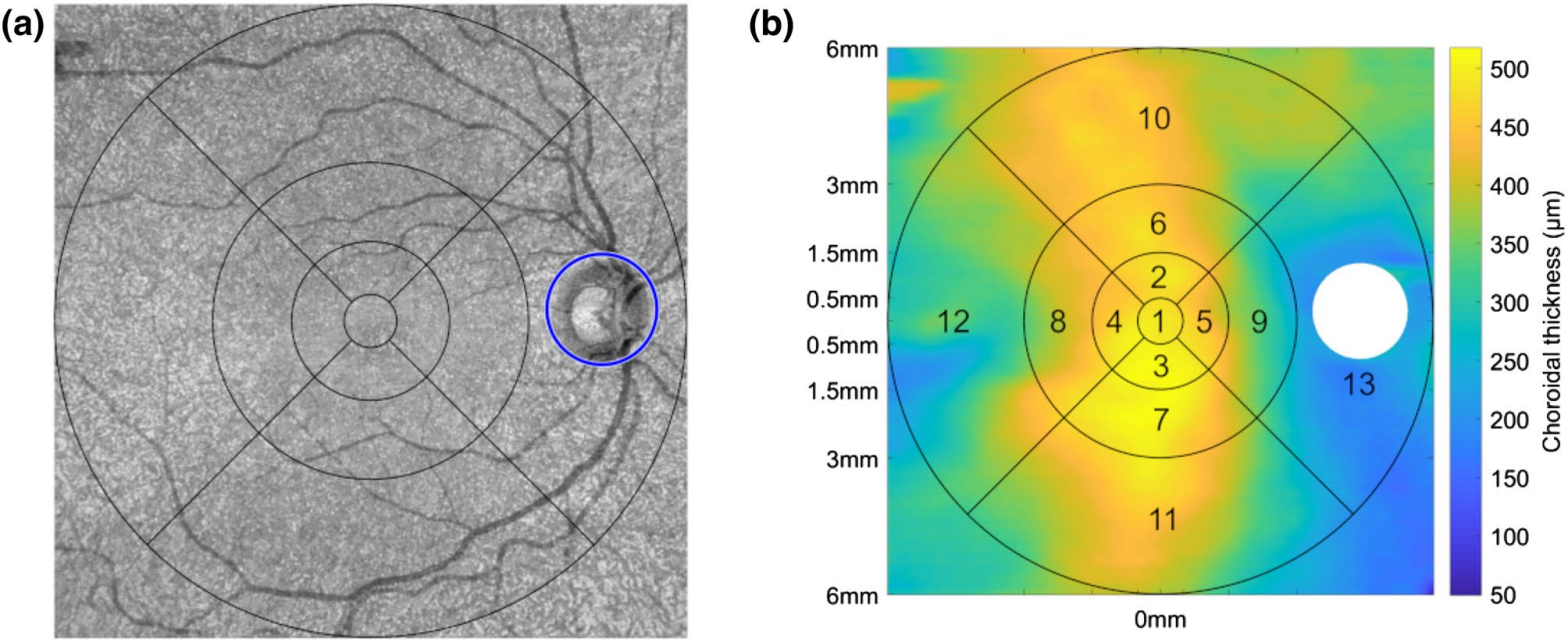
(**a**) Automatic detection of the location of the optic nerve head and visualization of ETDRS sectors on the en-face scan image of an example participant. (**b**) Extracted choroidal thickness map of the same participant together with the 13 wide-field ETDRS areas and the excluded thickness data from the optic nerve head.

Choroidal thickness was defined as the distance from the RPE to the chorio-scleral interface. Thickness maps were obtained from automated image segmentation via the Advanced Retinal Imaging Network (ARI Network, Carl Zeiss Meditec Inc., Dublin, CA, USA). The ARI Network represents a research portal provided by Carl Zeiss Meditec Inc., which offers various algorithms for researchers using the PlexElite 9000 OCT device. Thickness data from the optic nerve head was excluded in all maps due to layer segmentation errors. The location of the optic nerve head was automatically detected from the en-face images and directly transferred onto the choroidal thickness map, as seen in Fig. 4a. The resulting thickness maps were then converted from pixel to microns by a conversion factor of 1.9531, which is based on the fraction of true scan depth in microns to the image scan depth in pixels. Subsequently, the full scan area was divided into 13 extended wide-field Early Treatment of Diabetic Retinopathy Study (ETDRS)^45^ sectors with radii of 0.5 mm, 1.5 mm, 3 mm and 6 mm, as depicted in Fig. 4b. The median thickness value within a ETDRS region was chosen as the representative thickness value of the particular ETDRS area.

### Data analysis

Retinal shape calculation, foveal pit fitting, processing of the retinal and choroidal thickness maps and further statistical data analysis were performed in MATLAB (MATLAB 2019a, The MathWorks, Inc., Natick, MA, USA). Due to the partial non-parametric distribution of the investigated parameters, absolute values are given as median ± IQR. In case of retinal radius of curvature and foveal pit morphology, the ratios between horizontal and vertical measurement angles were additionally calculated, in order to find potential growth asymmetries between meridians. Spearman correlation was used to analyze the correlation coefficient $\rho$ between the retinal parameters and axial length and spherical refractive error. *p* values *p* < 0.05 were considered as significant.

## Data Availability

The datasets generated during and/or analysed during the current study are available from the corresponding author on reasonable request.
